# Public Reporting of Quality and Clinical Outcomes in the Get With The Guidelines–Stroke Registry

**DOI:** 10.1001/jamanetworkopen.2025.53244

**Published:** 2026-01-12

**Authors:** Michael T. Mullen, Juan Zhao, Tian Jiang, Zihang Gao, Jenny Buechner, Feras Wahab, Min Hee Seo, Ben Harder, Lee H. Schwamm, Gregg C. Fonarow, Eric E. Smith, Ying Xian, Steven R. Messé

**Affiliations:** 1 Lewis Katz School of Medicine, Temple University, Philadelphia, Pennsylvania; 2 American Heart Association, Dallas, Texas; 3 US News & World Report, Washington, DC; 4 Yale University School of Medicine, Yale University, New Haven, Connecticut; 5 Ronald Reagan UCLA Medical Center, University of California, Los Angeles; 6 Hotchkiss Brain Institute, University of Calgary, Calgary, Alberta, Canada; 7 Univerity of Texas Southwestern Medical Center, Dallas; 8 Perelman School of Medicine, University of Pennsylvania, Philadelphia; 9 Associate Section Editor, *JAMA Cardiology*

## Abstract

**Importance:**

Public reporting of outcomes increases transparency. Research on the association between public reporting programs and quality of care and outcomes is needed.

**Objective:**

To evaluate whether hospital voluntary participation in the Get With The Guidelines (GWTG)–Stroke public reporting program is associated with quality of care and clinical outcomes.

**Design, Setting, and Participants:**

This cohort study used GWTG-Stroke registry data from January 1 to December 31, 2021. Clinical characteristics associated with hospital participation in public reporting were identified, and differences in patient quality of care and outcomes were compared between hospitals that participated in public reporting of outcomes and those that did not. The primary data analysis was completed on December 27, 2024, and revised November 15, 2025.

**Exposure:**

Hospital participation in public reporting.

**Main Outcomes and Measures:**

The primary quality metric was defect-free care, a composite of 7 ischemic stroke quality measures (intravenous thrombolysis for patients arriving by 3.5 hours and treated by 4.5 hours, early antithrombotic use within 48 hours of admission, venous thromboembolism prophylaxis, antithrombotics at hospital discharge, anticoagulation for atrial fibrillation or flutter, smoking cessation counseling, and intensive statin therapy at discharge). Component measures were evaluated as secondary outcomes. The primary clinical outcome was independent ambulation at discharge. Secondary clinical outcomes included discharge to home, in-hospital mortality, and a composite of in-hospital mortality or discharge to hospice. Associations were measured using multivariable models adjusted for patient- and hospital-level variables.

**Results:**

There were 501 763 patients admitted for acute ischemic stroke (mean [SD] age, 69.8 [3.8] years; mean [SD] male, 51.5% [10.3%]) at 2423 hospitals; 1582 hospitals (65.3%) participated in public reporting. High-volume hospitals (quartile 4 [highest] vs 1 [lowest]: adjusted odds ratio [OR], 2.07 [95% CI, 1.43-2.99]) and high-performing hospitals measured by 2018 GWTG-Stroke quality awards (silver or gold: OR, 3.32 [95% CI, 2.63-4.20]) were more likely to participate in public reporting. In fully adjusted models, patients treated at participating hospitals were more likely to receive defect-free care (OR, 1.31 [95% CI, 1.27-1.35]) and more likely to receive all 7 of the individual components of defect-free care, although absolute differences between groups were small. There were minor differences in independent ambulation at discharge (OR, 1.02 [95% CI, 1.01-1.04]) and the composite of in-hospital mortality or discharge to hospice (OR, 1.05 [95% CI, 1.02-1.08]). There were no differences in discharge to home or in-hospital mortality.

**Conclusions and Relevance:**

In this cohort study of hospitals in the GWTG-Stroke program in 2021, patients at hospitals participating in voluntary public reporting were more likely to receive guideline-based care but had similar clinical outcomes compared with patients at nonreporting hospitals. More research is needed to evaluate whether public reporting could improve clinical outcomes.

## Key Points

**Question:**

What is the association of hospital participation in a quality registry voluntary public reporting program with quality of care and clinical outcomes?

**Findings:**

In this cohort study of 501 763 patients at 2423 hospitals participating in the Get With The Guidelines–Stroke registry, 65.3% of hospitals were participating in voluntary public reporting 2 years after the start of the program. Patients at participating hospitals were more likely to receive guideline-based care compared with those at nonparticipating hospitals, although clinical outcomes were similar.

**Meaning:**

These findings suggest that patients at public reporting hospitals may be more likely to receive guideline-based care; more research is needed to determine whether public reporting could improve outcomes.

## Teaser

This cohort study evaluates whether voluntary public reporting among Get With The Guidelines (GWTG)–Stroke hospitals is associated with quality of care and clinical outcomes.

## Introduction

Public reporting of health care outcomes has been proposed as a method of improving health care quality and lowering cost.^1,2^ Public reporting facilitates transparency. Accessible data on comparative quality has the potential to influence health care decisions at all levels, including patients, employers, payers, health care professionals, health care institutions, and policy makers.^3,4,5^ This should incentivize participating hospitals to improve the quality of care that they deliver, but existing data on the association of public reporting with quality and outcomes vary across programs.^5,6^ There is a need for further research detailing hospital participation in public reporting programs and quantifying the association between participation in public reporting programs and quality of care and clinical outcomes.

Get With The Guidelines (GWTG)–Stroke is a hospital-based quality improvement initiative and clinical registry developed by the American Heart Association more than 20 years ago.^7,8^ The registry is now used in more than 2000 hospitals and captures more than 80% of incident strokes in the US.^9^ Hospitals participating in the GWTG-Stroke registry receive awards based on adherence to quality measures. Participation in GWTG-Stroke has been associated with improvements over time in quality of care and outcomes.^8,10^ In August 2019, GWTG-Stroke announced a voluntary public reporting program centered around 7 stroke quality measures. In November 2019, *US News & World Report* (*USNWR*) announced that participating hospitals would receive a transparency credit in the subsequent year’s adult neurology and neurosurgery specialty ranking.^11^ Participating hospitals’ quality data are freely accessible on the American Heart Association’s Quality Near Me website, which displays summary data on quality measures based on an entire calendar year of performance.

Within the GWTG-Stroke registry, we aimed to (1) describe participation in the voluntary public reporting program, (2) identify hospital characteristics associated with participation in public reporting, and (3) evaluate whether there are differences in quality of care and clinical outcomes for patients treated at public reporting hospitals compared with those at nonreporting hospitals. We hypothesized that teaching and academic hospitals, large hospitals, high-volume hospitals, and high-performing hospitals would be more likely to participate in public reporting. We also hypothesized that patients treated at public reporting hospitals would be more likely to receive guideline-based care and have better clinical outcomes than patients treated at nonreporting hospitals.

## Methods

This cohort study included patients hospitalized with acute ischemic stroke at US hospitals participating in the GWTG-Stroke registry during calendar year 2021. Participating hospitals received either human research approval to enroll cases without individual patient consent under the Common Rule or a waiver of authorization and exemption from subsequent review by their institutional review board. The study followed the Strengthening the Reporting of Observational Studies in Epidemiology (STROBE) reporting guideline.

### Data Source

The GWTG-Stroke registry is an in-hospital program for improving stroke care by promoting adherence to evidence-based stroke management guidelines in the US. Participating hospitals record clinical characteristic, treatment, and outcome data of patients admitted for an acute ischemic stroke via a web-based patient management tool. IQVIA Inc is the data collection and coordination center. The GWTG-Stroke program has been described previously.^8,10^ There is a high degree of accuracy and concordance between data in the registry and medical record review.^12^

This study used data from all admissions with a final diagnosis of acute ischemic stroke between January 1 and December 31, 2021. We excluded patients with missing race as recorded in the GWTG-Stroke database (Asian, Black, White, other [American Indian or Alaska Native and Native Hawaiian or Pacific Islander], or unable to determine), Hispanic ethnicity, sex, and discharge disposition. Race and ethnicity were examined as there are well-described differences in outcome and treatment for racial and ethnic minority patients with stroke. For each hospital in GWTG-Stroke, we identified whether they participated in the public reporting program; the date they opted in; and, if applicable, the date they opted out. Data on hospital participation in public reporting were obtained from the American Heart Association. Data on hospital ranking in the *USNWR *neurology and neurosurgery rankings were obtained from *USNWR*. Hospital performance was characterized by whether the hospital received a quality award from GWTG-Stroke for the calendar year 2018, the year prior to the start of public reporting. The GWTG-Stroke awards are categorized as no award, bronze, silver, or gold (defined as ≥85% compliance in all 7 stroke achievement measures for 90 consecutive days [bronze], 12 consecutive months [silver], and 24 consecutive months [gold]).^13^

### Statistical Analysis

The primary analysis was completed on December 27, 2024, and revised on November 15, 2025, using R, version 4.2.0 (R Foundation for Statistical Computing). Statistical significance was assessed at 2-tailed α = .05. Univariable comparisons were performed using the 2-proportions *z* test for categorical variables and Kruskal-Wallis test for continuous variables. For baseline characteristics, absolute standardized differences were calculated as a measure of effect size between groups. An absolute standardized difference greater than 0.1 is considered a meaningful difference.^14^ Variable missingness is summarized in the eTable 1 in Supplement 1. Multiple imputation by chained equations was used for imputing missing National Institutes of Health Stroke Scale (NIHSS) scores. Outcome missingness is summarized in eTable 2 in Supplement 1. Outcomes were reported and analyzed only among complete cases.

For aim 1, we sought to describe the characteristics of hospitals that chose to participate in GWTG-Stroke public reporting. We quantified the proportion of GWTG-Stroke hospitals participating in public reporting at each of the first 5 opt-in dates for the program. Multivariable generalized linear models were used to identify hospital-level variables independently associated with public reporting. These models were adjusted for hospital-level variables, including quartile of stroke admissions per year, teaching status (academic or teaching, nonacademic or teaching), geographic region (West, Midwest, South, Northeast), hospital ownership (private, nonprofit, government), GWTG-Stroke 2018 quality award (no award, bronze, silver, gold), and The Joint Commission (TJC) stroke center designation (none, acute stroke ready center, primary stroke center, thrombectomy-capable stroke center, comprehensive stroke center). Hospital bed size was excluded from the model due to collinearity with stroke admissions per year, and rural and urban locations were excluded due to collinearity with stroke center certification.

For aim 2, we sought to evaluate whether patients treated at GWTG-Stroke hospitals participating in public reporting were more or less likely to receive guideline-based care than those treated at GWTG-Stroke hospitals not participating in public reporting. The primary outcome was defect-free care, a composite of the 7 publicly reported quality measures (intravenous thrombolysis for patients arriving by 3.5 hours and treated by 4.5 hours, early antithrombotic use within 48 hours of admission, venous thromboembolism prophylaxis, antithrombotics at hospital discharge, anticoagulation for atrial fibrillation or flutter, smoking cessation counseling, and intensive statin therapy at discharge). The secondary outcomes included each of the individual quality achievement measures. Among patients treated with intravenous thrombolysis, we also evaluated for differences in door-to-needle treatment within 30 minutes and within 60 minutes.

For aim 3, we sought to evaluate whether patients treated at GWTG-Stroke hospitals participating in public reporting had better or worse clinical outcomes than patients treated at GWTG-Stroke hospitals not participating in public reporting. The primary clinical outcome was independent ambulation at hospital discharge. To reduce confounding, the analysis was restricted to patients who were able to ambulate independently prior to admission. Secondary outcomes included discharge to home, in-hospital mortality (all cause), and the composite of in-hospital mortality (all cause) or discharge to hospice. Discharge modified Rankin Scale scores were not evaluated due to high rates of missingness (41.8%).

For aims 2 and 3, multivariable models were adjusted for patient characteristics, including age; sex; race and Hispanic ethnicity; NIHSS score; arrival mode; evening or weekend arrival; ambulatory status prior to admission; prestroke modified Rankin Scale score; medical history of hypertension, atrial fibrillation or flutter, coronary artery disease or prior myocardial infarction, carotid stenosis, diabetes, peripheral vascular disease, dyslipidemia, heart failure, prior stroke, drug or alcohol abuse, kidney insufficiency, and deep vein thrombosis or pulmonary embolism; and tobacco use, and for hospital characteristics, including quartile of stroke admissions per year, teaching status, geographic region, hospital ownership, and TJC stroke center designation. In an exploratory analysis, the models were recreated using generalized estimating equations (GEEs), which are robust to account for within-hospital clustering effects.

## Results

**Figure 1.**
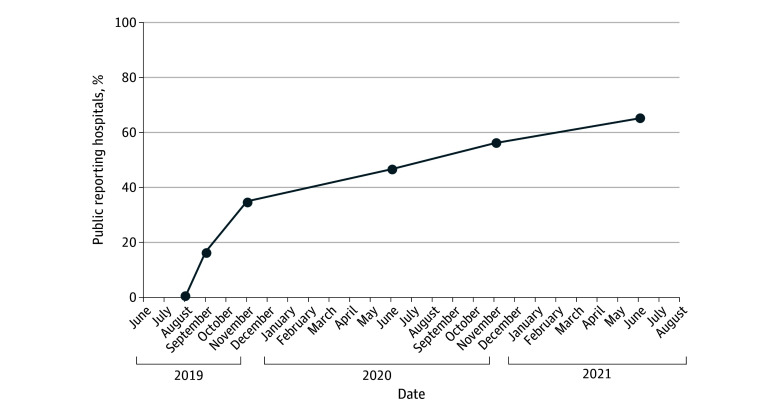
Trend of Hospital Participation in Get With The Guidelines–Stroke Public Reporting

There were 501 763 patients admitted for acute ischemic stroke (mean [SD] age, 69.8 [3.8] years; mean [SD] sex, 48.5% [10.3%] female and 51.5% [10.3%] male; mean [SD] race, 3.0% [7.2%] Asian, 15.5% [18.8%] Black, 69.3% [25.4%] White, 0.8% [3.9%] other, and 3.8% [8.3%] unable to determine; mean [SD] Hispanic ethnicity, 7.6% [13.9%]) from 2423 hospitals included in GWTG-Stroke during the study period. Of those 2423 hospitals, 1582 (65.3%) participated in public reporting. Figure 1 shows the proportion of hospitals participating in public reporting, which increased over time. Participation more than doubled after *USNWR* announced that public reporting hospitals would receive a transparency credit in its rankings program in October 2019.^11^

Table 1 summarizes aggregated patient- and hospital-level characteristics. There were small differences in patient-level characteristics, including age, race, Hispanic ethnicity, and mode of hospital arrival. Comparisons of insurance status were limited by missing data. Public reporting hospitals compared with nonreporting hospitals were larger (mean [SD] beds, 297.4 [260.6] vs 195.4 [183.3]), more commonly admitted patients transferred from another hospital (mean [SD] percentage of patients, 10.2% [13.9%] vs 6.1% [11.5%]), had more stroke admissions per year (mean [SD], 245.1 [203.0] vs 135.5 [159.9]), and were more frequently teaching institutions (1079 [68.2%] vs 409 [48.6%]). Public reporting hospitals also were more likely to be nonprofit (1137 [71.9%] vs 478 [56.8%]), located in urban areas (1252 [79.1%] vs 517 [61.5%]), located in the Northeast (284 [18.0%] vs 77 [9.2%]) and West (316 [20.0%] vs 128 [15.2%]), and certified as a stroke center by TJC (1000 [63.2%] vs 371 [44.1%]) and less likely to be located in the South (496 [31.4%] vs 365 [43.4%]). Public reporting hospitals had been participating in GWTG longer (mean [SD], 7.8 [6.0] vs 6.3 [5.3] years) and were higher performing based on receiving GWTG gold, silver, or bronze quality awards in 2018 (1208 [76.4%] vs 317 [37.7%]). Public reporting hospitals were also more likely to be a 2021 *USNWR* neurology and neurosurgery top-50 hospital (48 [3.0%] vs 0).

**Table 1.** Characteristics of Hospitals Participating in GWTG-Stroke Public Reporting and the Patients Treated at Those Hospitals, Aggregated Across Sites

Characteristic	Total (2423 hospitals)	Public reporting		ASD
		Yes (1582 hospitals)	No (841 hospitals)	
**Patient level (N = 501 763)**				
Age, mean (SD), y	69.8 (3.8)	70.0 (3.3)	69.5 (4.6)	0.152
Sex, mean (SD), %				
Female	48.5 (10.3)	48.6 (7.5)	48.2 (14)	0.062
Male	51.5 (10.3)	51.4 (7.5)	51.8 (14.0)	0.062
Race, mean (SD), %				
Asian	3.0 (7.2)	3.4 (7.2)	2.2 (7.1)	0.162
Black	15.5 (18.8)	15.9 (18.4)	14.7 (19.4)	0.069
White	69.3 (25.4)	68.2 (24.3)	71.3 (27.3)	0.127
Other^a^	0.8 (3.9)	0.8 (3.5)	0.8 (4.4)	0.004
Unable to determine	3.8 (8.3)	3.8 (7.7)	3.7 (9.2)	0.013
Hispanic ethnicity, mean (SD), %	7.6 (13.9)	7.8 (12.7)	7.3 (15.9)	0.044
Insurance, mean (SD), %				
Medicaid	12 (11.5)	12.5 (10.9)	11.1 (12.6)	0.127
Medicare	36.5 (23.4)	37.9 (22)	33.7 (25.6)	0.193
Private, VA, TRICARE (formerly CHAMPUS), or other	26.6 (19.2)	28.4 (18.2)	23.4 (20.5)	0.275
Self-pay or no insurance	4 (6.1)	3.8 (5.4)	4.5 (7.4)	0.133
Not documented	20.9 (35.9)	17.4 (33.3)	27.3 (39.7)	0.299
NIHSS score, mean (SD)^b^	5.9 (2.3)	5.9 (1.9)	6.0 (2.7)	0.027
Arrival mode, mean (SD), %				
EMS	45.9 (16.6)	46.2 (14.9)	45.3 (19.4)	0.058
Private transport	41.2 (17.6)	39.6 (16)	44.3 (20)	0.289
Transfer from another hospital	8.8 (13.3)	10.2 (13.9)	6.1 (11.5)	0.296
Mobile stroke unit	0.1 (0.7)	0.1 (0.8)	0 (0.5)	0.091
Not documented or unknown	0.8 (3.9)	0.8 (3.5)	1 (4.7)	0.049
Missing	3.2 (7.6)	3.1 (5.7)	3.3 (10.2)	0.042
Intravenous thrombolysis DTN time				
Mean (SD), min^c^	63.7 (21.6)	60.9 (17.8)	69.4 (27.1)	0.475
≤30 min, mean (SD), %^c^	12.7 (16.9)	14 (16.7)	10.0 (16.9)	0.240
≤60 min, mean (SD), %^c^	59.2 (26.8)	62.6 (24.1)	52.2 (30.5)	0.430
**Hospital level**				
No. of hospital beds				
Mean (SD)	262.4 (241.7)	297.4 (260.6)	195.4 (183.3)	0.392
Median (range)	200 (5-2911)	227 (6-2911)	151 (5-1414)	
Ischemic stroke admissions per year				
Mean (SD)	207.1 (196.2)	245.1 (203)	135.5 (159.9)	0.540
Median (range)	156 (1-1458)	191 (1-1458)	86 (1-1218)	
Perform EVT, No. (%)	616 (25.4)	498 (31.5)	118 (14.0)	0.376
Teaching status, No. (%)				
Major	214 (8.8)	188 (11.9)	26 (3.1)	0.272
Minor	1274 (52.6)	891 (56.3)	383 (45.5)	0.217
Nonteaching	738 (30.5)	381 (24.1)	357 (42.4)	0.430
Missing	197 (8.1)	122 (7.7)	75 (8.9)	0.045
Hospital location				
Rural	455 (18.8)	208 (13.1)	247 (29.4)	0.435
Urban	1769 (73.0)	1252 (79.1)	517 (61.5)	0.480
Missing	199 (8.2)	122 (7.7)	77 (9.2)	0.054
Hospital ownership				
Government	285 (11.8)	166 (10.5)	119 (14.1)	0.119
Nonprofit	1615 (66.7)	1137 (71.9)	478 (56.8)	0.334
Private	325 (13.4)	156 (9.9)	169 (20.1)	0.343
Missing	198 (8.2)	123 (7.8)	75 (8.9)	0.043
Region				
Midwest	559 (23.1)	364 (23.0)	195 (23.2)	0.004
Northeast	361 (14.9)	284 (18.0)	77 (9.2)	0.220
South	861 (35.5)	496 (31.4)	365 (43.4)	0.260
West	444 (18.3)	316 (20.0)	128 (15.2)	0.119
Missing	198 (8.2)	122 (7.7)	76 (9.0)	0.050
TJC stroke center designation				
Acute stroke	80 (3.3)	43 (2.7)	37 (4.4)	0.103
Comprehensive	216 (8.9)	187 (11.8)	29 (3.4)	0.259
Primary	983 (40.6)	697 (44.1)	286 (34.0)	0.203
Thrombectomy capable	92 (3.8)	73 (4.6)	19 (2.3)	0.112
Not designated	1052 (43.4)	582 (36.8)	470 (55.9)	0.396
Quality award received in 2018				
Gold	1290 (53.2)	1036 (65.5)	254 (30.2)	0.742
Silver	191 (7.9)	141 (8.9)	50 (5.9)	0.104
Bronze	44 (1.8)	31 (2.0)	13 (1.5)	0.030
None	898 (37.1)	374 (23.6)	524 (62.3)	0.910
No. of years participating in GWTG				
Mean (SD)	7.3 (5.8)	7.8 (6.0)	6.3 (5.3)	0.251
Missing	43 (1.8)	2 (0.1)	41 (4.9)	0.360
*USNWR* 2021 neurology and neurosurgery ranking				
Top 50 hospitals	48 (2.0)	48 (3.0)	0	0.177
Not top 50 hospitals	2375 (98.0)	1534 (97.0)	841 (100.0)	0.177

Abbreviations: ASD, absolute standardized difference; CHAMPUS, Civilian Health and Medical Program or the Uniformed Services; DTN, door to needle; EMS, emergency medical service; EVT, endovascular thrombectomy; GWTG, Get With The Guidelines; NIHSS, National Institutes of Health Stroke Scale; TJC, The Joint Commission; *USNWR*, *US News & World Report*; VA, US Department of Veterans Affairs.

^a^ Other includes American Indian or Alaska Native and Native Hawaiian or Pacific Islander.

^b^ Range from 0 to 42, with higher scores indicating more severe stroke symptoms.

^c^ Process metrics were calculated among eligible patients, excluding those with documented contraindications.

**Figure 2.**
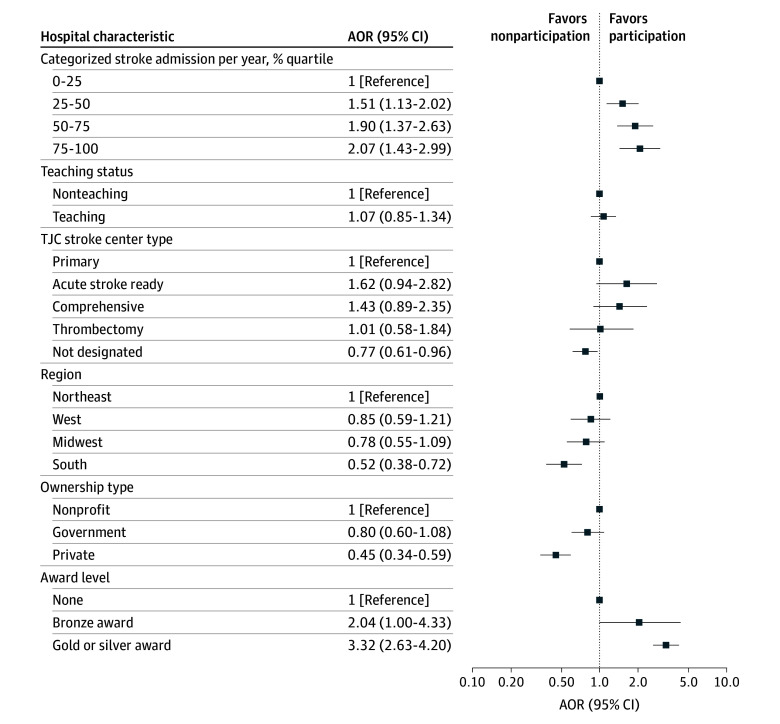
Association of Hospital-Level Variables and Participation in Public Reporting of Outcomes AOR indicates adjusted odds ratio; TJC, The Joint Commission.

Multivariable model results are presented in Figure 2. Hospital characteristics associated with an increased odds of public reporting included stroke admissions per year compared with quartile 1 (quartile 2: adjusted odds ratio [AOR], 1.51 [95% CI, 1.13-2.02; *P* = .001]; quartile 3: AOR, 1.90 [95% CI, 1.37-2.63; *P* < .001]; quartile 4: AOR, 2.07 [95% CI, 1.43-2.99; *P* < .001]) and a 2018 GWTG gold or silver quality award (AOR, 3.32 [95% CI, 2.63-4.20]; *P* < .001). Variables that were associated with a decreased odds of public reporting included absence of stroke center certification by TJC (AOR, 0.77 [95% CI, 0.61-0.96]; *P* = .02), hospital location in the South (AOR, 0.52 [95% CI, 0.38-0.72]; *P* < .001), and private hospital ownership (AOR, 0.45 [95% CI, 0.34-0.59]; *P* < .001).

Table 2 summarizes quality measure performance for patients at public reporting and nonreporting hospitals. Defect-free care was more frequent at participating hospitals (patients with the outcome, 95.2% vs 92.3%; unadjusted OR, 1.64 [95% CI, 1.60-1.69]; *P* < .001). All 7 of the individual quality measures were more frequent at public reporting hospitals, although absolute differences were generally small. In the fully adjusted models, which included patient- and hospital-level characteristics, defect-free care (OR, 1.31 [95% CI, 1.27-1.35]; *P* < .001), intravenous thrombolysis arrival by 3.5 hours and treated by 4.5 hours (OR, 1.60 [95% CI, 1.49-1.71]; *P* < .001), early antithrombotics (OR, 1.13 [95% CI, 1.08-1.19]; *P* < .001), venous thromboembolism prophylaxis (OR, 1.45 [95% CI, 1.34-1.57]; *P* < .001), antithrombotics at discharge (OR, 1.15 [95% CI, 1.05-1.25]; *P* = .002), anticoagulation for atrial fibrillation or flutter (OR, 1.24 [95% CI, 1.11-1.39]; *P* < .001), smoking cessation (OR, 1.44 [95% CI, 1.28-1.61]; *P* < .001), and intensive statin therapy at discharge (OR, 1.27 [95% CI, 1.16-1.39]; *P* < .001) remained significantly more likely at public reporting hospitals. Patients treated with intravenous thrombolysis at public reporting hospitals were also more likely to be treated with a door-to-needle time less than 60 minutes (OR, 1.15 [95% CI, 1.10-1.21]; *P* < .001) and less than 30 minutes (OR, 1.22; 95% CI, 1.15-1.29]; *P* < .001). In the GEE analysis (eTable 3 in Supplement 1), the results were broadly similar, although early antithrombotics and antithrombotics at discharge were not significantly different between groups.

**Table 2.** Association Between Hospitalization at a Facility That Participates in Public Reporting and Care Quality Metrics

Quality outcome	Patients with the outcome, No./total No. (%)		Unadjusted model		Fully adjusted model^a^	
	Public reporting	Nonreporting	OR (95% CI)	*P* value	OR (95% CI)	*P* value
Defect-free care	301 481/316 693 (95.2)	85 597/92 695 (92.3)	1.64 (1.60-1.69)	<.001	1.31 (1.27-1.35)	<.001
IV thrombolysis arrival by 3.5 h and treated by 4.5 h	37 742/40 467 (93.3)	11 349/13 209 (85.9)	2.27 (2.13-2.42)	<.001	1.60 (1.49-1.71)	<.001
IV tPA among patients arriving within 4.5 h						
DTN ≤30 min	8532/39 933 (21.4)	2054/11 895 (17.3)	1.30 (1.23-1.37)	<.001	1.22 (1.15-1.29)	<.001
DTN ≤60 min	28 387/39 933 (71.1)	7829/11 895 (65.8)	1.28 (1.22-1.33)	<.001	1.15 (1.10-1.21)	<.001
Early antithrombotics	189 652/19 603 (96.7)	56 945/59 387 (95.9)	1.27 (1.22-1.34)	<.001	1.13 (1.08-1.19)	<.001
VTE prophylaxis	239 363/24 134 (99.2)	67 036/68 088 (98.5)	1.90 (1.76-2.05)	<.001	1.45 (1.34-1.57)	<.001
Antithrombotics at discharge	272 955/274 871 (99.3)	78 127/78 971 (98.9)	1.54 (1.42-1.67)	<.001	1.15 (1.05-1.25)	.002
Anticoagulation for atrial fibrillation or flutter	46 034/47 221 (97.5)	12 075/12 586 (95.9)	1.64 (1.48-1.82)	<.001	1.24 (1.11-1.39)	<.001
Smoking cessation^b^	51 928/52 905 (98.2)	14 953/15 478 (96.6)	1.87 (1.68-2.08)	<.001	1.44 (1.28-1.61)	<.001
Intensive statin therapy at discharge	203 390/205 073 (99.2)	57 866/58 631 (98.7)	1.60 (1.47-1.74)	<.001	1.27 (1.16-1.39)	<.001

Abbreviations: DTN, door to needle; IV, intravenous; OR, odds ratio; tPA, tissue plasminogen activator; VTE, venous thromboembolism.

^a^ Generalized linear models were adjusted for patient characteristics, including age; sex; race and Hispanic ethnicity; National Institutes of Health Stroke Scale score at admission; arrival mode; evening or weekend arrival; ambulatory status prior to admission; prestroke modified Rankin Scale score; medical history of atrial fibrillation or flutter, coronary artery disease or prior myocardial infarction, hypertension, carotid stenosis, diabetes, peripheral vascular disease, dyslipidemia, heart failure, prior stroke, drug or alcohol abuse, kidney insufficiency, deep vein thrombosis or pulmonary embolism, and hypertension; and tobacco use, and for hospital characteristics, including quartile of stroke admissions per year, teaching status, geographic region, hospital ownership, and The Joint Commission stroke center designation.

^b^ Tobacco use was not included for smoking cessation as only patients with active tobacco use were included in this metric.

Table 3 summarizes clinical outcomes for patients at public reporting and nonreporting hospitals. In the fully adjusted models, the odds of independent ambulation at hospital discharge were higher at public reporting hospitals (OR, 1.02 [95% CI, 1.01-1.04]; *P* = .007). In the fully adjusted models, the odds of the composite in-hospital mortality or discharge to hospice was higher at public reporting hospitals (OR, 1.05 [95% CI, 1.02-1.08]; *P* < .001). There were no differences in discharge to home or in-hospital mortality in the adjusted models. In the GEE analysis (eTable 4 in Supplement 1), there were no significant differences in any of the outcomes.

**Table 3.** Association Between Hospitalization at a Facility That Participates in Public Reporting and Clinical Outcomes

Clinical outcome	Patients with the outcome, No./total No. (%)		Unadjusted model		Fully adjusted model^a^	
	Public reporting	Nonreporting	OR (95% CI)	*P* value	OR (95% CI)	*P* value
Independent ambulation at hospital discharge	123 884/212 070 (58.4)	33 942/57 596 (58.9)	0.98 (0.96-1.00)	.03	1.02 (1.01-1.04)	.007
Discharge to home	182 122/363 737 (50.1)	54 170/108 911 (49.7)	1.01 (1.00-1.03)	.06	1.01 (0.99-1.03)	.20
In-hospital mortality	16 017/363 737 (4.4)	4045/108 911 (3.7)	1.19 (1.15-1.24)	<.001	0.98 (0.95-1.02)	.43
Composite of in-hospital mortality or discharge to hospice	33 698/363 737 (9.3)	8926/108 911 (8.2)	1.14 (1.12-1.17)	<.001	1.05 (1.02-1.08)	.001

Abbreviation: OR, odds ratio.

^a^ Generalized linear models were adjusted for patient characteristics, including age; sex; race and Hispanic ethnicity; National Institutes of Health Stroke Scale score at admission; arrival mode; evening or weekend arrival; ambulatory status prior to admission; prestroke modified Rankin Scale score; medical history of atrial fibrillation or flutter, coronary artery disease or prior myocardial infarction, hypertension, carotid stenosis, diabetes, peripheral vascular disease, dyslipidemia, heart failure, prior stroke, drug or alcohol abuse, kidney insufficiency, deep vein thrombosis or pulmonary embolism, and hypertension; and tobacco use, and for hospital characteristics, including quartile of stroke admissions per year, teaching status, geographic region, hospital ownership, and The Joint Commission stroke center designation.

## Discussion

This cohort study found that by June 2021 of the GWTG-Stroke voluntary public reporting program, 65.3% of hospitals chose to participate and patients at public reporting hospitals were more likely to receive defect-free care and received each of the 7 publicly reported quality measures. While public reporting of outcomes should have an inherent incentive effect, our results suggest that high-volume stroke hospitals and high-performing hospitals were more likely to participate in public reporting. Observed differences in this study may reflect the broader success of the GWTG-Stroke program, which is associated with improved performance on quality measures over time, than the public reporting program.^8,10^

Patients had similar clinical outcomes at public reporting and nonreporting hospitals. There was a statistically significant increase in independent ambulation at hospital discharge at public reporting hospitals, suggesting slightly better patient outcomes. However, there was also a statistically significant increase in the composite of in-hospital mortality or discharge to hospice, suggesting slightly worse outcomes in patients at public reporting hospitals. Importantly, the absolute differences for all outcomes were small. There were no differences in discharge to home or in-hospital mortality in the primary analysis. There were no differences in any outcomes in the GEE analysis (eTable 4 in Supplement 1). Taken as a whole, these data suggest broadly similar outcomes at public reporting and nonreporting hospitals.

There are several factors that may have contributed to the overall similar outcomes between groups. The GWTG-Stroke public reporting program centers on quality measures and does not include discharge outcomes. For the most part, these quality measures are not expected to have a causal link to the discharge outcomes measured in this study. During the study period, all hospitals were subject to mandatory reporting of 30-day risk-adjusted mortality and rehospitalization through the Centers for Medicare & Medicaid Services Hospital Quality Initiative and, so, were equally incentivized to improve on those metrics. Second, there may be residual bias and confounding that influenced these outcomes. Hospitals that participated in public reporting were larger and more commonly admitted patients as a transfer from another hospital (10.2% vs 6.1%). It is possible that patients at participating hospitals were more severely injured, despite similar NIHSS scores. Finally, GWTG-Stroke participation has been associated with improved clinical outcomes at all hospitals, regardless of participation in voluntary public reporting.^15,16^ Future study is needed to investigate whether participation in GWTG-Stroke and public reporting could lead to greater improvements in outcomes over time compared with participation in GWTG-Stroke alone.

Public reporting programs have proliferated in recent years. Data have suggested that they are associated with improved quality and outcomes, although these may vary substantially based on the specific program, disease state, and outcomes being reported.^5,17,18,19,20,21,22,23^ For public reporting to be meaningful, the data must be accurate, clinically relevant, and actionable. Failure to meet these requirements could limit the impact of a program. For example, a recent survey of referring physicians’ perspectives on the Society for Thoracic Surgeries Congenital Heart Disease public reporting program found that while 83% felt that public reporting was important, 60% had doubts about the accuracy of the information reported, and 92% stated that public reporting data would rarely or never override other factors in determining referrals.^24^ In another survey of interventional cardiologists in New York State, 90% did not feel that mortality statistics were an accurate measure of physician quality, and 75% did not believe that the state angioplasty quality report served to improve care.^25^

Data from the GWTG-Stroke public reporting program come from a hospital-based quality improvement registry that has been shown to be reliable and valid.^12^ However, our findings highlight several challenges that are relevant for policy makers. First, our results suggest that when participation is voluntary, there may be differential participation across hospitals. Because high-performing hospitals were more likely to participate, publicly available quality reports do not accurately reflect the full range of hospital performance. Hospitals that appear to be performing below average based on publicly available data may still outperform nonreporting hospitals. We also found lower participation in the South, despite a high burden of cerebrovascular disease in that region.^26^ While we are unable to speculate why hospitals in the South were less likely to participate, this finding highlights that voluntary public reporting programs could lead to disparate data availability across regions or communities. Second, the overall rates of adherence to the 7 reported quality measures were very high, which may speak to the success of the GWTG-Stroke program. However, with such high rates, there may be too little variation across facilities for clinically meaningful differentiation. Third, although patients at public reporting hospitals were more likely to receive guideline-based care than patients at nonparticipating hospitals, clinical outcomes may be of greater relevance to public health. Direct reporting of clinical outcomes could potentially improve the utility of the GWTG-Stroke public reporting program, although appropriate risk adjustment is required to account for case-mix differences across hospitals. Stroke is an unplanned, time-critical illness. As such, public reporting may be more relevant to payers and public health professionals interested in improving regional stroke systems of care than to patients and caregivers. It may be that data elements such as length of stay, patient satisfaction scores, and financial metrics are more relevant to those groups. Further research engaging patients, payers, and public health professionals to identify the optimal data points for stroke public reporting would be useful.

### Limitations

This study had several limitations. We could not assess for a causal relationship between public reporting and outcomes, and as noted, high-volume and high-performing hospitals were more likely to participate in public reporting. This study used a single year of data from 2021. As a rapidly evolving field, it is possible that results using more recent data would be different. We could not determine whether participation in public reporting was associated with improvements in care over time and should be assessed in future work using additional years of data. The GWTG-Stroke registry relies on hospital-reported outcomes. Differences in outcome ascertainment or reporting across hospitals has the potential to bias the results. Multiple imputation was used for missing NIHSS scores. It is possible that the data were not missing at random, and so potential for bias remains. Clinical outcomes were evaluated at hospital discharge. Stroke discharge outcomes have been correlated with longer-term outcomes, but 30- or 90-day outcomes may provide a more accurate assessment.^27^

## Conclusions

In this cohort study of data from the GWTG-Stroke public reporting program, hospital participation was commonplace soon after the program’s inception. High-volume hospitals and high-performing hospitals, as measured by GWTG-Stroke quality awards, were more likely to participate in public reporting. Patients treated at public reporting hospitals were more likely to receive defect-free care and meet individual GWTG-Stroke quality metrics, although absolute differences were generally small. Clinical outcomes were broadly similar for patients at public reporting and nonreporting hospitals. Further studies are needed to optimize stroke outcome reporting and investigate whether public reporting contributes to improved stroke quality and outcomes over time.

## Notes

Supplement 1. **eTable 1.** Variable Missingness **eTable 2.** Missing Counts for Quality and Clinical Outcomes **eTable 3.** Association Between Hospitalization at a Facility That Participates in Public Reporting and Quality Outcomes Using GEE Models **eTable 4.** Association Between Hospitalization at a Facility That Participates in Public Reporting and Clinical Outcomes Using GEE Models

Supplement 2.
Data Sharing Statement
