# Supplementary material for: Public Reporting of Quality and Clinical Outcomes in the Get With The Guidelines–Stroke Registry
Source: JAMA Netw Open. 2026 Jan 12;9(1):e2553244. doi: 10.1001/jamanetworkopen.2025.53244 (PMC12797097; doi:10.1001/jamanetworkopen.2025.53244)
Supplement: Supplement 1. — eTable 1. Variable Missingness eTable 2. Missing Counts for Quality and Clinical Outcomes eTable 3. Association Between Hospitalization at a Facility That Participates in Public Reporting and Quality Outcomes Using GEE Models eTable 4. Association Between Hospitalization at a Facility That Participates in Public Reporting and Clinical Outcomes Using GEE Models [file jamanetwopen-e2553244-s001.pdf]

Supplemental Online Content

Mullen MT, Zhao J, Jiang T, et al. Public reporting of quality and clinical outcomes in the Get With The Guidelines–Stroke registry. *JAMA Netw Open*. 2026;9(1):e2553244. doi:10.1001/jamanetworkopen.2025.52344

eTable 1. Variable Missingness

eTable 2. Missing Counts for Quality and Clinical Outcomes

eTable 3. Association Between Hospitalization at a Facility That Participates in Public Reporting and Quality Outcomes Using GEE Models

eTable 4. Association Between Hospitalization at a Facility That Participates in Public Reporting and Clinical Outcomes Using GEE Models

This supplemental material has been provided by the authors to give readers additional information about their work.

© 2026 Mullen MT et al. JAMA Network Open.

eTable 1. Variable Missingness

**Co-variates**	**Missing rate (%)**	**Notes**
Hospital participated in the public reporting or not	0	
Age	0	
Gender	0	
Race	0	
NIHSS score at admission	6.3	Applied Multivariate Imputation by Chained Equations (MICE)
Arrival mode	3.1	Assigned the mode 'EMS from home/scene'
Arrive Off hours	0	
Prior ambulatory status	25.5	Assigned 'Unable to determine'
Pre-stroke modified Rankin score	37.0	Assigned 'Unable to determine'
Thrombolytics initiated at the hospital or not	0.9	Assigned 'No'
Medical history Atrial fib or flutter	1.4	Assigned 'No'
Medical history CAD or prior MI	1.4	Assigned 'No'
Hypertension	1.4	Assigned 'No'
Medical history Carotid Stenosis	1.4	Assigned 'No'
Medical history diabetes mellitus	1.4	Assigned 'No'
Medical history PVD	1.4	Assigned 'No'
Medical history dyslipidemia	1.4	Assigned 'No'
Medical history heart failure	1.4	Assigned 'No'
Medical history previous stroke	1.4	Assigned 'No'
Medical history drug alcohol abuse	1.4	Assigned 'No'
Medical history renal insufficiency	1.4	Assigned 'No'
Medical history DVT/PE	1.4	Assigned 'No'
Medical history hypertension	1.4	Assigned 'No'

© 2026 Mullen MT et al. JAMA Network Open.

eTable 2. Missing Counts for Quality and Clinical Outcomes

Quality outcome	N with the outcome	N not eligible	N missing the outcome
Defect-free care (composite measure)	431314	70449	0
IV thrombolysis arrive by 3.5 hours, treat by 4.5 hours	56780	444983	0
IV tPA among patients arriving within 4.5h, Treat by 4.5 Hour - DTN3 ≤30 min	54834	446929	0
IV tPA among patients arriving within 4.5h, Treat by 4.5 Hour - DTN3 ≤60 min	54834	446929	0
Antithrombotics at discharge	375163	126600	0
Anticoagulation for Afib/Aflutter	63053	438710	0
Smoking cessation	72227	429536	0
Intensive statin at discharge	279631	222132	0
**Clinical Outcome**	**N with the outcome**	**N not eligible**	**N missing the outcome**
Modified Rankin scale 0-2 at discharge	291967	0	209796
Independent ambulation at hospital discharge	432674	0	69089
Discharge to home	501763	0	0
In-hospital mortality	501763	0	0
Composite of in-hospital mortality or hospice discharge	501763	0	0

© 2026 Mullen MT et al. JAMA Network Open.

eTable 3. Association Between Hospitalization at a Facility That Participates in Public Reporting and Quality Outcomes Using GEE Models

	Unadjusted model (GEE)		Fully adjusted model (GEE)	
**Quality outcome**	OR (95% CI)	p-value	OR (95% CI)	p-value
Defect-free Care (composite measure)	1.64 (1.48 - 1.83)	<0.001	1.31 (1.17 - 1.45)	<0.001
IV thrombolysis arrive by 3.5 hours, treat by 4.5 hours	2.27 (1.85 - 2.78)	<0.001	1.60 (1.29–1.98)	<0.001
IV tPA among patients arriving within 4.5h - DTN ≤30 min	1.30 (1.08 - 1.57)	0.006	1.22 (1.02–1.45)	0.03
IV tPA among patients arriving within 4.5h - DTN ≤60 min	1.28 (1.14 - 1.43)	<0.001	1.15 (1.04–1.28)	0.005
Early antithrombotics	1.27 (1.11 - 1.46)	<0.001	1.13 (0.97- 1.31)	0.12
VTE prophylaxis	1.90 (1.56 - 2.31)	<0.001	1.45 (1.18 - 1.78)	<0.001
Antithrombotics at discharge	1.54 (1.17 – 2.02)	0.002	1.15 (0.84 - 1.57)	0.38
Anticoagulation for Afib/Aflutter	1.64 (1.40 - 1.92)	<0.001	1.24 (1.06 - 1.45)	0.008
Smoking cessation	1.87 (1.49 - 2.34)	<0.001	1.44 (1.16 - 1.79)	0.001
Intensive statin at discharge	1.60 (1.33 - 1.92)	<0.001	1.27 (1.04 - 1.55)	0.02

© 2026 Mullen MT et al. JAMA Network Open.

eTable 4. Association Between Hospitalization at a Facility That Participates in Public Reporting and Clinical Outcomes Using GEE Models

	**Unadjusted model (GEE)**		**Fully adjusted Model (GEE)**	
**Clinical Outcome**	OR (95% CI)	p-value	OR (95% CI)	p-value
Independent ambulation at hospital discharge	0.98 (0.88- 1.08)	0.68	1.02 (0.92 - 1.14)	0.66
Discharge to home	1.01 (0.98 - 1.05)	0.48	1.01 (0.97 - 1.05)	0.61
In-hospital mortality	1.19 (1.09 - 1.31)	<0.001	0.98 (0.91- 1.07)	0.70
Composite of in-hospital mortality or hospice discharge	1.14 (1.08 - 1.21)	<0.001	1.05 (1.00- 1.11)	0.06

© 2026 Mullen MT et al. JAMA Network Open.
