# Supplementary material for: Public Reporting of Quality and Clinical Outcomes in the Get With The Guidelines–Stroke Registry
Source: JAMA Netw Open. 2026 Jan 12;9(1):e2553244. doi: 10.1001/jamanetworkopen.2025.53244 (PMC12797097; doi:10.1001/jamanetworkopen.2025.53244)
Supplement: Supplement 2. — Data Sharing Statement [file jamanetwopen-e2553244-s002.pdf]

Data Sharing Statement

Data
Data available: No

Additional Information
Explanation for why data not available: American Heart Association data is collected for clinical care and quality improvement, rather than primarily for research. Data sharing agreements require an application process for other researchers to access the data.
